# Household-level risk factors for *Aedes aegypti* pupal density in Guayaquil, Ecuador

**DOI:** 10.1186/s13071-021-04913-0

**Published:** 2021-09-07

**Authors:** Thien-An Ha, Tomás M. León, Karina Lalangui, Patricio Ponce, John M. Marshall, Varsovia Cevallos

**Affiliations:** 1 School of Public Health, University of California, Berkeley, USA; 2 Centro de Investigación en Vectores Artrópodos, Instituto Nacional de Investigación en Salud Pública “Dr. Leopoldo Izquieta Pérez”, Quito, Ecuador

**Keywords:** *Aedes aegypti*, Mosquito, Household risk factors, Arbovirus, Collection services, Precipitation, Predictive modeling

## Abstract

**Background:**

Vector-borne diseases are a major cause of disease burden in Guayaquil, Ecuador, especially arboviruses spread by *Aedes aegypti* mosquitoes. Understanding which household characteristics and risk factors lead to higher *Ae. aegypti* densities and consequent disease risk can help inform and optimize vector control programs.

**Methods:**

Cross-sectional entomological surveys were conducted in Guayaquil between 2013 and 2016, covering household demographics, municipal services, potential breeding containers, presence of *Ae. aegypti* larvae and pupae, and history of using mosquito control methods. A zero-truncated negative binomial regression model was fitted to data for estimating the household pupal index. An additional model assessed the factors of the most productive breeding sites across all of the households.

**Results:**

Of surveyed households, 610 satisfied inclusion criteria. The final household-level model found that collection of large solid items (e.g., furniture and tires) and rainfall the week of and 2 weeks before collection were negatively correlated with average pupae per container, while bed canopy use, unemployment, container water volume, and the interaction between large solid collection and rainfall 2 weeks before the sampling event were positively correlated. Selection of these variables across other top candidate models with ∆AICc < 1 was robust, with the strongest effects from large solid collection and bed canopy use. The final container-level model explaining the characteristics of breeding sites found that contaminated water is positively correlated with *Ae. aegypti* pupae counts while breeding sites composed of car parts, furniture, sewerage parts, vases, were all negatively correlated.

**Conclusions:**

Having access to municipal services like bulky item pickup was effective at reducing mosquito proliferation in households. Association of bed canopy use with higher mosquito densities is unexpected, and may be a consequence of large local mosquito populations or due to limited use or effectiveness of other vector control methods. The impact of rainfall on mosquito density is multifaceted, as it may both create new habitat and “wash out” existing habitat. Providing services and social/technical interventions focused on monitoring and eliminating productive breeding sites is important for reducing aquatic-stage mosquito densities in households at risk for *Ae. aegypti*-transmitted diseases.

**Graphical Abstract:**

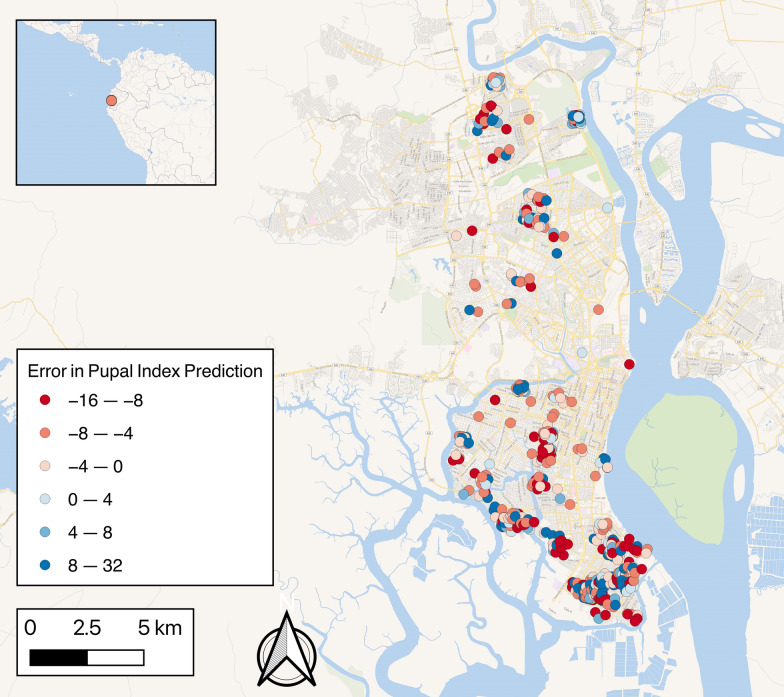

**Supplementary Information:**

The online version contains supplementary material available at 10.1186/s13071-021-04913-0.

## Background

Vector-borne febrile illnesses such as dengue, chikungunya, and Zika virus are of pressing public health concern in Latin America and the Caribbean [1]. The mosquito *Aedes aegypti* is the region’s primary vector of these arboviruses, which co-circulate in populations in the tropics and subtropics [1, 2]. The burden of these diseases weighs heavily on susceptible populations in low- and middle-income countries such as Ecuador [1].

Between 2010 and 2014, over 70,000 cases of dengue were reported in Ecuador, with the highest incidence clustered in urbanized coastal areas like the city of Guayaquil [1, 3]. Dengue infection can be asymptomatic or present as a moderate febrile illness, with some symptoms advancing to hemorrhage, shock, and death [4]. Without an effective dengue vaccine, community and household-level vector control of *Ae. aegypti* remains the primary means of preventing and controlling dengue outbreaks [2]. In Ecuador, each household currently employs, on average, five different mosquito control methods, including sprays, aerosols, repellents, mosquito coils, screens, and bed nets [1].

*Aedes aegypti* population management is an ongoing public health challenge for countries with limited resources that must efficiently plan and utilize targeted control. *Aedes aegypti* is a mosquito species that primarily amplifies epidemics among urban populations [5]. The species is an effective vector for dengue because it is highly adapted to urban environments, where it lays eggs in artificial containers of water near human dwellings and preferentially feeds on humans [6]. Adult *Ae. aegypti* lay eggs in such habitats, and larvae develop in both natural water-retaining structures and in domestic water containers [7]. Examples of outdoor breeding sites for *Ae. aegypti* include large tires, flower vases, and plastic gallon containers [8]. Understanding the local characteristics of *Ae. aegypti* habitats can be used to inform vector control efforts [9]. Previous studies done in Machala, Ecuador, found that local socio-ecological conditions such as proximity to abandoned properties, interruptions in the piped water supply, and a highly shaded patio were risk factors for *Ae. aegypti* proliferation and the presence of dengue [2]. Further investigation into household factors, in conjunction with the evaluation of vector control efforts, is necessary to reduce and prevent dengue incidence by reducing *Ae. aegypti* habitat and population.

Our study describes potential household-level risk factors for *Ae. aegypti* pupal proliferation in the city of Guayaquil, Ecuador’s largest and most populous city, its most important commercial port, and the historical epicenter of yellow fever and dengue in the country [1].

## Methods

### Study site

**Fig. 1:**
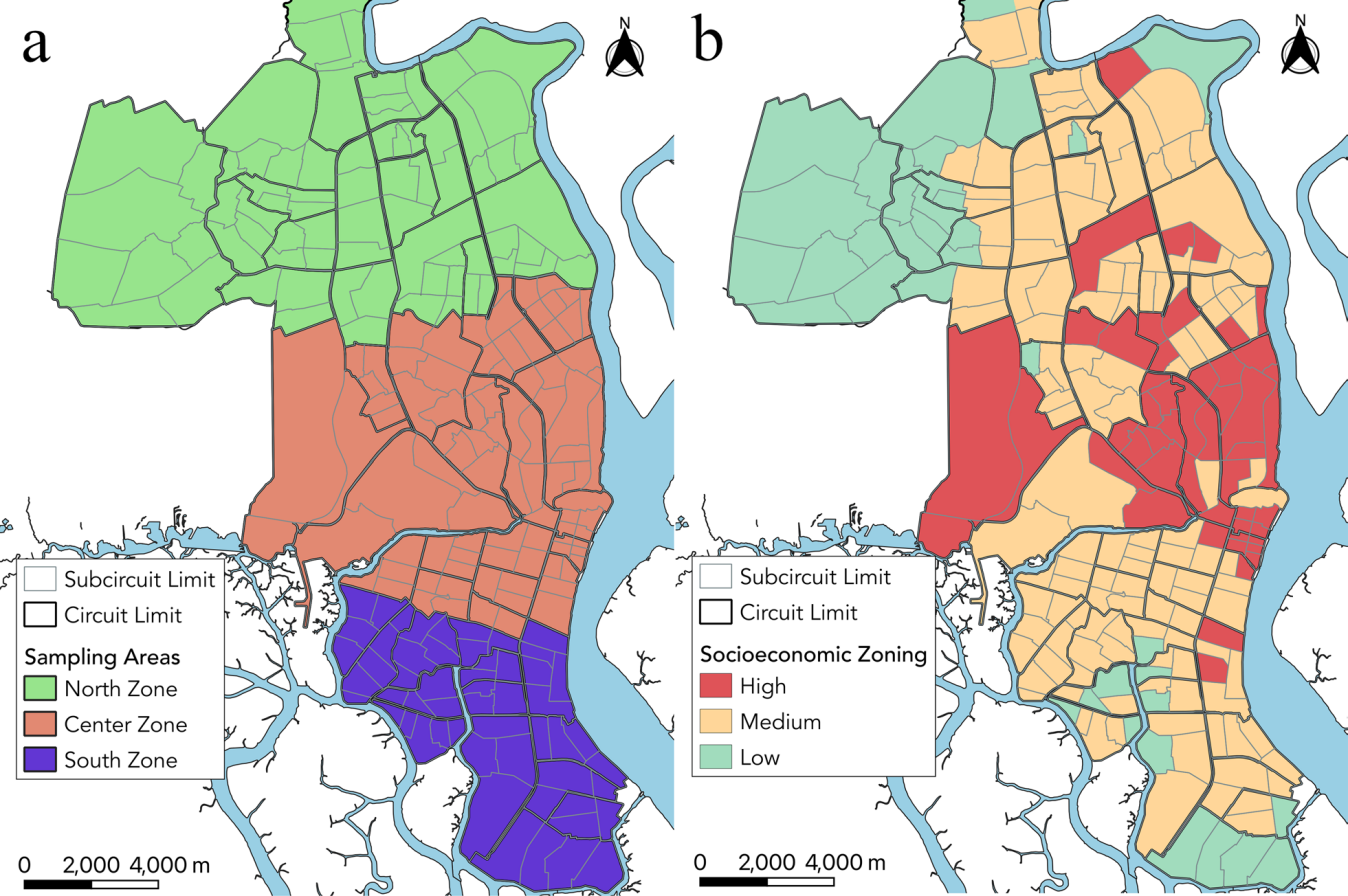
Subcircuits and zones of Guayaquil showing sampling zones (**a**) and socioeconomic status (**b**) by subcircuit

Guayaquil (2016 Instituto Nacional de Investigación en Salud Pública [INISP] projected population: 2,482,789) is located on the west bank of the Guayas River (Fig. 1), which flows into the Pacific Ocean. The urban core of Guayaquil is surrounded by low-income neighborhoods with limited basic services and high rates of migration. Guayaquil has had the greatest number of dengue cases in Ecuador since 1988, with all four serotypes circulating since then. Seasonally, the highest incidence occurs in the rainy season because of favorable environmental conditions for transmission. The first 4 months of the year have abundant rain in this coastal region, with over 17 average days of rain totaling more than 200 mm each month; in the province of Guayas, the days are hot and humid, with average high temperatures between 29 and 32 °C and high humidity (US National Oceanic and Atmospheric Administration Integrated Surface Database [NOAA ISD] data).

### Household data collection

Household cross-sectional surveys were conducted every month in Guayaquil from January 2013 to August 2016 to investigate factors correlated with mosquito pupal counts per container. Each month, one random subcircuit (an administrative unit covering ~ 10,000 inhabitants) was randomly selected in the Northern, Central, and Southern zones of Guayaquil, resulting in three sampling events (Fig. 1A). Guayaquil subcircuits were also classified into low, medium, and high socioeconomic status derived from three indicators: illiteracy, overcrowding, and unemployment (Fig. 1B). Each of these variables were averaged across the subcircuit, where illiteracy and unemployment were represented as percentages and overcrowding was the average number of people per household.

Households were defined as in-use residential units. Household addresses were obtained prior to the survey. Containers were defined as in-use breeding sites near or inside the household. Each container was examined for immature mosquito stages in the water, material, and type. Each house was visited only once in order to maximize the geographical area covered. Each visit assessed mosquito presence and conducted sampling in artificial containers of water. During each house visit, questions were asked to any household member over the age of 18 after verifying their residency. These questions focused on *Ae. aegypti* risk factors, including Ministry of Health vector control efforts and mosquito control practices which were used as candidate predictors in our model (Table 1, Additional file 1: Table S1).

**Table 1:** Summary statistics for habitat and vector control effort variables included in the analysis

Variables of interest	Number of households (*n* = 610)	Variable description
Number of children		Number of children (< 18 years) residing in the household
0	172 (28%)	
1–3	373 (61%)	
4–7	61 (10%)	
> 7	4 (1%)	
Number of adults		Number of adults (≥ 18 years) residing in the household
0–3	323 (53%)	
4–7	258 (42%)	
> 7	29 (5%)	
Water interruption		Whether or not the household experience a water service interruption in the last 24 h
Yes	153 (25%)	
No	457 (75%)	
Trash service per week		Number of times garbage collections occurs at the household per week
0–3	529 (87%)	
4–7	81 (13%)	
Large solid collection		Municipal service of collecting large furniture, tires, or other items
Yes	478 (78%)	
No	132 (22%)	
Sewer connection		Whether or not there is a sewer connection to the household’s waste system
Yes	550 (90%)	
No	60 (10%)	
Fumigation		Spraying of deltamethrin, an insecticide applied inside the house every four months
Yes	190 (31%)	
No	420 (69%)	
Abate		Also known as temephos, an organophosphate larvicide which is applied by 20 g of granular product per 189 L
Yes	151 (25%)	
No	459 (75%)	
Biolarvicide		*Bacillus thuringiensis* used to target the larval stage of a mosquito
Yes	322 (53%)	
No	288 (47%)	
Canopy use		The usage of a canopy over a bed
Yes	287 (47%)	
No	323 (53%)	
Protective mesh		Mesh present around doors and windows
Yes	90 (15%)	
No	520 (85%)	
Avg. water volume (L)		Total water volume in all household breeding containers divided by the number of breeding sites
1–25	435 (71%)	
26–50	21 (4%)	
> 50	154 (25%)	

### Meteorological data collection

Precipitation data were obtained from the high-resolution satellite of the Climate Prediction Center (CMORPH), with a temporal frequency every 0.5 h and a spatial resolution of 8 × 8 km^2^. Precipitation data every 0.5 h were then aggregated by epidemiological week.

Precipitation measurements at week 0, week 1 lag, and week 2 lag were included. Week 0 indicates the amount of rainfall the week that the sampling event occurred. The lag variables, week 1 lag and week 2 lag, indicate precipitation 1 week previous to the sampling event and precipitation 2 weeks previous to the sampling event, respectively. Only variables that were most relevant to mosquito ecology based on literature review and entomologist consultation were included for the models.

### Entomological sample collection

Three field technicians conducted each sampling event, which took place across 250 households over a period of 5 days each time monthly during the study period (2013–2016). The criteria to select the sampling areas were based on areas with a high number of dengue cases reported by the Ecuadorian Health Ministry (MSP 2020). Sampling was done in all the neighborhoods (Northern, Central, and Southern zones) at the same time. Homes were chosen at random using the neighborhood main street as the transect. If there was no response in the selected home, a nearby home was selected. In each house, technicians searched for immature mosquitoes in containers both inside and outside of the household. Each container carrying immatures was recorded for its container type and material type. These live immatures were transported to an insectary in Quito, where the lab at INSPI recorded the numbers and stage of development. Immatures were reared until the adult stage for full species identification.

### Statistical analysis

All analyses were performed in R version 3.5.3. We used zero-truncated negative binomial models to assess the appropriate household-level and container-level predictors of *Ae. aegypti* pupal population. Zero-truncated models were used because the data was only recorded for containers with immature mosquitoes present. Our outcome for the household-level statistical models was average pupal counts per container (APC), or pupal index, and was calculated as the total number of household pupae divided by the number of containers carrying these immature-stage mosquitoes. Our outcome for the secondary analysis on container-level data was the sum of the pupae in each artificial breeding site. We determined that outliers for each outcome were those beyond Q3 (75th percentile) + 1.5 * IQR (Q3 − Q1) and omitted them [10].

We performed a Chi-square test for the Poisson model assumption that conditional variance equals conditional mean in our data set. We rejected the null hypothesis that the Poisson model best fits our data (*P* < 0.01) and fitted a negative binomial model with a dispersion parameter of 1.4103 and a standard error of 0.0881. We fitted a full model using all variables for the household model (Additional file 1: Table S1) and the container model (Additional file 2: Table S2) and used the ‘dredge’ function (R package MuMIn v1.43.17) to find all possible models through the best subset selection technique [11]. Best subset selection exhaustively searches all combinations of candidate variables and ranks models using specific selection criteria. In this study, we used the Akaike information criterion corrected for small sample sizes (AICc) to compare all the candidate models (Additional file 5: Fig S1 for the household-level models and Fig S2 for the container-level model). AICc has a penalty term for small sample sizes: as sample size increases, AIC is approximated, and therefore AICc is preferred over AIC [12]. Effect sizes were considered significant if 95% confidence intervals for corresponding explanatory variables did not overlap zero. We evaluated model performances using 100 simulations of tenfold cross-validation as our model evaluation method.

## Results

In the surveys, 830 households of the total 990 households surveyed were found to have *Ae. aegypti* mosquito pupae. Of the 830 households, 220 had missing or erroneous location data (e.g., coordinates indicated a household was not in Guayaquil) and were omitted, yielding 610 households for our analysis. The mean pupal index for the subset of included households was 11.08, with a standard deviation of 10.26, and a maximum pupal index of 42.

About 47% of the 610 households used bed canopies as a method for preventing mosquito biting (Table 1). Approximately 25% of households had water service interruptions. Only 22% of households did not have large solid collection services. About 15% of households had protective mesh around their windows and doors.

### Influence of household-related factors on *Ae. aegypti* pupal abundance

The model with the smallest (best) AICc value included the variables canopy use, large solid service, unemployment, water volume, precipitation at week 0, precipitation at week 2 lag, and the interaction between large solid service and precipitation at week 2 lag (Table 2). The predictors with statistically significant associations were consistently selected into our top models ∆AICc < 1 (Table 2). The explanatory variable estimates for the model with the smallest AICc value, our top model, indicated that canopy use, unemployment, average container water volume, and the interaction between large solid service and precipitation (with 2-week lag) all had a statistically significant positive relationship with *Ae. aegypti* pupal abundance and large solid service had a significant negative relationship with *Ae. aegypti* pupal abundance (Table 3).

**Table 2:** Variables included in models with the smallest (best) AICc values

Model	*df*	LogLik	ΔAICc	Error
Canopy use + large solid service + unemployment + water volume + precipitation_0_ + precipitation_2_ + large solid service * precipitation_2_	9	−2075.162	0	10.08
Canopy use + water interruption + large solid service + unemployment + water volume + precipitation_0_ + precipitation_2_ + water interruption * precipitation_0_ + large solid service * precipitation_2_	11	−2073.210	0.2374	10.12
Canopy use + large solid service + unemployment + water volume + precipitation_2_ + large solid service * precipitation_2_	8	−2076.444	0.5036	10.10
Biolarvicide + canopy use + large solid service + unemployment + water volume + precipitation_0_ + precipitation_2_ + large solid service * precipitation_2_	10	−2074.407	0.5566	10.09
Biolarvicide + canopy use + water interruption + large solid service + unemployment + water volume + precipitation_0_ + precipitation_2_ + water interruption * precipitation_0_ + large solid service * precipitation_2_	12	−2072.431	0.7603	10.13

Only models that lie within a ∆AICc of 1 of the smallest AICc value are shown. The response variable was the total number of household *Ae. aegypti* pupae over the total number of household breeding sites. Error indicates the cross-validation prediction error off of the mean pupal index of 11.08. Precipitation_0_ indicates rainfall from the week of sampling and precipitation_2_ indicates rainfall with 2-week lag (i.e., 2 weeks before sampling)

**Table 3:** Explanatory variable estimates for the model with the smallest AICc value

Variable	Log estimate	Estimate	95% CI
Intercept	1.790	6.00	(3.68, 9.79)
Canopy use	0.240	1.271*	(1.06, 1.52)
Large solid service	−0.280	0.756*	(0.610, 0.932)
Unemployment	0.0641	1.0662*	(1.02, 1.114)
Water volume	0.00166	1.0016*	(1.0003, 1.003)
Precipitation_0_	−0.00600	0.994	(0.986, 1.0013)
Precipitation_2_	−0.0103	0.990	(0.970, 1.0118)
Large solid service * precipitation_2_	0.0214	1.0216*	(1.00, 1.043)

The response variable is the average pupae per container in a household

*P < 0.05

**Fig. 2:**
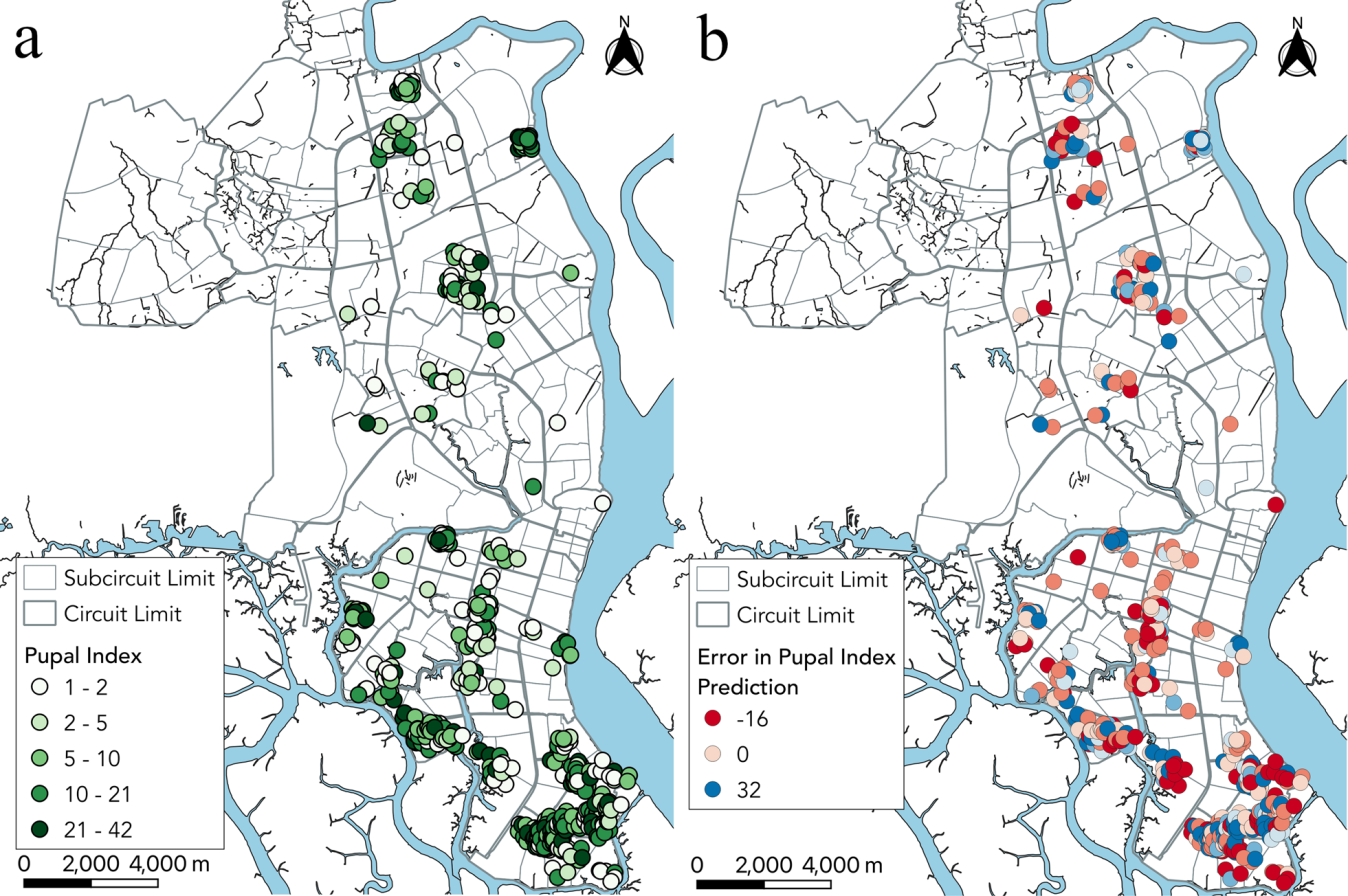
Pupal index measurements (**a**) and prediction error map (**b**) based on the final model. Each dot indicates a household included in the final analysis. Error is the difference between the data and the model for each household’s characteristics

The average prediction error over 100 simulations of tenfold cross-validation was 10 pupae off of the true value, where the mean pupal index was 11.08. Figure 2 shows the distribution of pupal index measurements in households and prediction error mapped across Guayaquil (also see Additional file 7: Fig. S3 for corresponding heat maps and Additional file 8: Fig. S4 for relative error). Because of the tightly clustered sampling of some neighborhoods and the contribution of local environmental effects, there was significant spatial autocorrelation (Moran’s *I* test, *P* < 0.01). The socioeconomic variables (such as unemployment) captured some of these effects, but there were likely other unmeasured exposures contributing to the spatial pattern. An assessment of multicollinearity, excluding interaction terms, revealed that in our top model, none of the variables tested had variance inflation factor scores above 2, indicating that there is little collinearity between predictors (Additional file 3: Table S3).

### Influence of container-related factors on *Ae. aegypti* pupal abundance

We used data from 924 containers to conduct our analysis. The mean pupal sum for the included containers was 15.46, with a standard deviation of 21.63, and a maximum pupal count of 253. We observed that the top container-level models (∆AICc < 1) consistently selected contaminated water, sewer parts, vases, ceramic material, glass material as appropriate predictors of pupal sum (Table 4, Additional file 4: Table S4).

**Table 4:** Variables included in container-level models with the smallest (best) AICc values

Model	*df*	LogLik	ΔAICc
Car parts + contaminated water + furniture + ceramic material + glass material + metal material + plastic material + sewer + vase	11	−3459.19	0
Car parts + contaminated water + ceramic material + glass material + metal material + plastic material + sewer + vase	10	−3460.23	0.03
Bamboo + car parts + contaminated water + furniture + ceramic material + glass material + metal material + plastic material + sewer + vase	12	−3458.20	0.08
Bamboo + car parts + contaminated water + ceramic material + glass material + metal material + plastic material + sewer + vase	11	−3459.24	0.11
Bucket part + car parts + contaminated water + ceramic material + glass material + metal material + plastic material + sewer + tub + vase	12	−3458.28	0.24

Only models that lie within a ∆AICc of 1 of the smallest AICc value are shown. The response variable was the total number of container-level *Ae. aegypti* pupae in each breeding site. Top five of all models with ∆AICc < 1 displayed

The average prediction error over 100 simulations of tenfold cross validation for the container models was 21.54 pupae off of the true mean value of 15.46 pupae. The variables car parts, sewer, vase, ceramic material, glass material, metal material, and plastic material were all found to have a significant negative association with the outcome, pupal sum (Table 5). Contaminated water was the singular predictor found to have a significant positive association with pupal sum (Table 5).

**Table 5:** Explanatory variable estimates for the container model with the smallest AICc value

Variable	Log estimate	Estimate	95% CI
Intercept	3.129	22.844	(17.74, 29.98)
Car parts	−0.666	0.513*	(0.35, 0.74)
Contaminated water	0.247	1.280*	(1.10, 1.49)
Furniture	−0.484	0.616	(0.35, 1.21)
Ceramic material	−0.942	0.390*	(0.23, 0.68)
Glass material	−0.900	0.407*	(0.23, 0.74)
Metal material	−0.425	0.654*	(0.47, 0.90)
Plastic material	−0.477	0.620*	(0.47, 0.81)
Sewer	−0.617	0.540*	(0.31, 1.02)
Vase	−0.414	0.661*	(0.46, 0.97)

Response variable is pupal sum per container

*P < 0.05

## Discussion

The burden of arboviruses transmitted by *Ae. aegypti* in Guayaquil has increased significantly since the 1980s, and targeted interventions are necessary to halt the spread of such diseases [3]. The findings of this study provide evidence that *Ae. aegypti* proliferation is influenced by specific household risk factors. The household models that performed best as determined by AICc contained the following variables: canopy use, large solid collection services, unemployment, water volume, water interruptions, biolarvicide, precipitation at week 0, precipitation at week 2 lag, and the interactions precipitation at week 0 * water interruption and large solid collection services * precipitation at a week 2 lag.

Canopy use was found to have a significant positive association with *Ae. aegypti* abundance, which is counterintuitive and may be attributed to other vector control practices being limited when bed canopies are in use. It is likely that those with higher mosquito populations inside the house are more prone to using bed canopies. The usage of these canopies may result in a false sense of security, or may be an indication of other unmeasured household risk factors that allow for high *Ae. aegypti* densities. The previously mentioned study from Machala, Ecuador, also cites an unclear relationship between dengue infections in a city near Guayaquil and bed canopy usage [2]. Furthermore, *Ae. aegypti* are daytime feeders, so these nets would only affect mosquito feeding if household members are napping during the day.

In our analysis, large solid collection services had a significant negative association with pupal abundance, which may be because there remain fewer untouched breeding sites available for *Ae. aegypti*. Regular bulky item pickup removes tires and other potential mosquito habitats where water could pool. This is corroborated by previous studies which have found that trash and flower pots are among the most common *Ae. aegypti*-positive containers [13]. These containers may be more regularly eliminated and maintained in higher socioeconomic areas through large solid collection services. In alignment with this finding, it was also found that unemployment had a significant positive relationship with pupal abundance. This corresponds with the comparison of the socioeconomic status map from Fig. 1 and the pupal index map from Fig. 2, where we find that there is a higher density of *Ae. aegypti*-positive households in lower socioeconomic areas. There is also a higher prediction error for these areas, as seen in Fig. 2 and Additional file 7: Fig. S3, suggesting that areas with higher unemployment need an emphasis on research to better understand the specific household risk factors attributed to *Ae. aegypti* pupal density (Additional file 6: Fig. S2).

Our study also found that average water volume had a significant positive relationship with pupal abundance. A 2012 study from the Tri Nguyen village in Vietnam found that containers where the water volume increased relative to the previous survey had a significantly higher count of *Ae. aegypti* pupae [14]. The study also found that the greatest increase in pupal abundance occurred after a rainfall event. This corresponds to our study’s findings in which both precipitation during week 0 and increasing water volume resulted in higher APC. Heavy rainfall is known to flush out existing containers, which could explain the negative (not statistically significant) association between rainfall during the week of sampling and APC. A negative association (although again not statistically significant) between rainfall with a 2-week lag and APC could conceivably result from the same flushing phenomenon, adjusting for the 8–12 days for *Ae. aegypti* eggs to develop into pupae [15, 16]. The interaction between large solid collection services and precipitation 2 weeks prior was significantly positively associated with *Ae. aegypti* pupal counts, which could be due to rainfall providing habitat for eggs to be laid which then develop into pupae 8–12 days later. The meaning of the interaction is somewhat unclear; however, wealthier neighborhoods had increased access to large solid collection services, so there was greater creation and destruction of mosquito habitat compared with poorer neighborhoods. Figure 2 shows that wealthier areas of Guayaquil have a lower number of high-density *Ae. aegypti* households. With fewer breeding habitats in wealthier neighborhoods, precipitation may have a larger and differential effect on pupal density, and therefore, any marginal effect may be picked up by the model.

This differentiation is further explained in the water storage practices and distribution of houses. In neighborhoods with higher employment rates and lower illiteracy, there are an increased number of natural areas where precipitation may collect, especially since houses are spread further apart. In less developed neighborhoods, there are different relationships with standing containers. During the rainy season, there are not as many water-holding containers because the water is constantly replenished by the rain. However, in the dry season, there are more standing containers because water is more scarce and needs to be stored for the households.

When the interaction term is included in the final model, precipitation at a week 2 lag has a negative correlation with the outcome. When the interaction term is not being controlled for, 14 days after a precipitation event correlates with higher pupal density. This suggests that there is a specific relationship between large solid collection services and precipitation at a week 2 lag on our outcome, pupal density. However, since large solid services may serve as a proxy for socioeconomic status, this may suggest a dynamic effect across socioeconomic statuses. These nuances are difficult to account for within the model context. For vector control efforts to be effective, it may require a more thorough understanding of the relationship between rainfall and socioeconomic factors that influence pupal density.

*Aedes aegypti* are highly adaptable mosquitoes that were historically found in forested areas using tree holes for breeding but have since adapted to breeding in tires, vases, and other objects found in proximity to human habitations [17]. Their resilience and adaptability pose difficulties when searching for effective control methods, especially for outdoor areas [17]. However, in light of our analyses, certain types and materials of containers may be more or less productive than others.

In our study, vase-type containers were found to be a significant predictor and were correlated with lower pupae counts. Glass material composition was selected as an appropriate explanatory predictor and was correlated with lower pupae counts as well. Vases, other glass-type containers, metal material, and ceramic material containers may have more variable water temperature that impedes *Ae. aegypti* development. Contaminated water was found to be a significant predictor correlated with higher pupae counts. The survey had field technicians qualitatively assess whether water was contaminated, so it was not quantifiably measured. Research suggests that *Ae. aegypti* prefer “clean” water, but this is a relative designation, as some nutrients in the water may support mosquito populations [7]. Contaminated water may have organic components within the container that promote algal growth and support mosquito proliferation. Water that is contaminated is likely to be untouched and stagnant, allowing *Ae. aegypti* to lay eggs and develop, as opposed to cleaner water, which may be flushed more often [7]. This finding is corroborated by previous studies that have noted that poor sanitation and water storing habits provide viable habitats for *Ae. aegypti* [3].

These results suggest that trash collection services targeting large solids and monitoring of containers that could serve as juvenile mosquito habitat contribute to suppressing *Ae. aegypti* pupal proliferation and consequent adult mosquito densities. These predictive models provide household factors of interest that could be included in future surveys to test hypotheses or assessed in rigorous causal models.

For the top household model, the mean error was high (10 pupae off of the true value) relative to the mean pupal index (11.0836). However, the standard deviation of the data is 10.263 indicating that the high error is due to the relatively high variance of the data, and the maximum pupal count is 253. Overdispersion and high variance are common in insect count data; therefore, these results remain valid [18].

There were months without sampling in each of the years for each of the three parts of Guayaquil; however, they did not share the same months missing in each area, so it was not possible to address this through a time-series analysis to account for the repeated measurements on households. Predictive modeling has limitations. Best subset selection assesses 2^*p*^ models, where *p* indicates the number of parameters, making the implementation of every interaction computationally infeasible when the number of parameters is large. Using previous literature, we assessed the most pertinent interactions and limited our model variable subset selection to 2^21^ models (Additional file 5: Fig. S1). This study could be improved with the inclusion of zero APC households to differentiate between containers and households that have zero mosquito pupae compared with those that have positive counts. Additionally, a longitudinal study, as opposed to the cross-sectional study design here, could track temporal dynamics in pupae populations. With a longitudinal study, a time-series analysis would be able to assess changing exposures to vector control methods and the environment and any subsequent changes in mosquito populations.

Future studies could correlate pupae counts with household demographics such as age and sex of inhabitants. Noting behavioral differences across these characteristics could also inform efforts for reducing mosquito proliferation and arbovirus spread. Additionally, further studies should compare our estimates of household factors in Guayaquil to those in more rural settings. Household risk factors such as water service interruption and temephos use may have a larger impact in more rural areas, where water interruptions may be more frequent. A similar study placed on an urban-to-rural gradient may help capture these effects. Additionally, dengue serological data could be incorporated to assess correlation between household risk factors and past exposure to dengue, which would be closer to the health endpoint and valuable for the public health sector. Random-effects modeling may further assess our covariates and outcomes with contextual understanding of variable distributions between and within households. Lastly, an understanding of competing dynamics between *Ae. aegypti* and other species of mosquitoes for habitat, breeding, and feeding would provide further context for targeted interventions in areas where multiple species coexist.

Furthermore, in this study, we used average pupae per container as our outcome measure. In this research field, similar studies have used other measures such as the Breteau index and house and container indices [1]. For this study, house and container indices were not used because the data set does not contain records of negative containers that did not contain *Ae. aegypti* larvae necessary to compute such indices. The Breteau index was not appropriate for this study because we explored individual household-level characteristics; however, this could be applied to a neighborhood-level study in the future.

## Conclusions

The results of this study indicate that household factors influenced *Ae. aegypti* pupae proliferation from 2013 to 2016 in Guayaquil, Ecuador. The most notable household-level risk factors for pupae proliferation were the use of bed canopies, unemployment, and water volume in artificial containers, as well as precipitation with 2-week lag in conjunction with large solid collection. Providing services and social/technical interventions focused on monitoring and eliminating breeding sites may be important for reducing aquatic-stage mosquito densities in households at risk for *Ae. aegypti*-transmitted diseases. The development of *Ae. aegypti* prediction models contributes to public health efforts in Ecuador by providing information to optimize interventions for reducing mosquito densities and preventing dengue outbreaks.

## Supplementary Information

**Additional file 1: Table S1.** Full list of household candidate variables used to find the best model by AICc.

**Additional file 2: Table S2.** Full list of container-level candidate variables used to find the best model by AICc.

**Additional file 3: Table S3.** Variance inflation factors (VIF) that assess multicollinearity find that (outside of interaction terms) there are no VIF scores > 2.

**Additional file 4: Table S4.** Full list of top candidate models for artificial breeding sites. Top models included have ∆AICc < 1.

**Additional file 5: Figure S1.** Model selection table where the top axis are all the candidate variables for pupal index (Additional file 1: Table S1) and the *y*-axis represents the frequency the variables were selected in the top candidate models with ∆AICc < 2 (31 models shown).

**Additional file 6: Figure S2.** Model selection table where the top axis are all the candidate variables for pupal sum in containers (Additional file 2: Table S2) and the *y*-axis represents the frequency the variables were selected in the top candidate models with ∆AICc < 1 (23 models total shown).

**Additional file 7: Figure S3.** Pupal index measurements (**a**) and prediction error heat maps (**b**) based on the final model. Weighted by value with a bandwidth of 1 km. Error is the difference between the data and the model for each household’s characteristics.

**Additional file 8: Figure S4.** Relative error map for pupal index prediction based on the final model. Each dot indicates the relative prediction error of that household, i.e., the difference between data and model for each household’s characteristics divided by the data.

## Data Availability

The data sets generated and analyzed during the current study are not publicly available as they are the property of the Centro de Investigación en Vectores Artrópodos, Instituto Nacional de Investigación en Salud Pública “Dr. Leopoldo Izquieta Pérez”, Quito, Ecuador. Data are however available from the corresponding author on reasonable request.

## Abbreviations

AIC: Akaike information criterion
AICc: Akaike information criterion corrected for small sample sizes
APC: Average pupal counts per container
IQR: Interquartile range
VIF: Variance inflation factors
