# Supplementary material for: Household-level risk factors for Aedes aegypti pupal density in Guayaquil, Ecuador
Source: Parasit Vectors. 2021 Sep 7;14:458. doi: 10.1186/s13071-021-04913-0 (PMC8425057; doi:10.1186/s13071-021-04913-0)
Supplement: Supplementary file 1 — Additional file 1: Table S1. Full list of household candidate variables used to find the best model by AICc. [file 13071_2021_4913_MOESM1_ESM.docx]

Table S1. Full list of household candidate variables used to find the best model by AICc.

| Full list of model variables | Variable description |
| --- | --- |
| Illiteracy | A relative subcircuit index on illiteracy |
| Unemployment | A relative subcircuit index on unemployment |
| Overcrowding | A relative subcircuit index on overcrowding |
| Number of children | Number of children (<18 years) residing in the household |
| Number of adults | Number of adults ( ≥ 18 years) residing in the household |
| Water interruption | Whether or not the household experience a water service interruption in the last 24 hours |
| Duration of water interruption | Duration of the water service interruption |
| Trash collection service | Number of times garbage collections occurs at the household per week |
| Large solid collection service | Municipal service of collecting large furniture, tires or other items |
| Sewer connection | Whether or not there is a sewer connection to the household’s waste system |
| Fumigation in past weeks | Spraying of deltamethrin, an insecticide applied inside the house in the last 1-4 weeks |
| Abate in past weeks | Also known as temephos, an organophosphate larvicide which is applied by 20 grams of granular product per 189 liters |
| Biolarvicide in past weeks | *Bacillus thuringiensis* used to target the larval stage of a mosquito |
| Bed canopy use | The usage of a canopy over a bed |
| Protective window/door mesh | Mesh present around doors and windows |
| Volume of breeding site | Average dimensions of household breeding sites |
| Water volume of breeding site | Total water volume in all household breeding containers divided by the number of breeding sites |
| Current dengue case | Household case of dengue at time of interview |
| Dengue in the last month | Household case of dengue within the last month at time of interview |
| Precipitation at week 0 | Rainfall during the week of the household interview |
| Week 1 precipitation lag | Rainfall during the week prior to the household interview |
| Week 2 precipitation lag | Rainfall two weeks prior to the household interview |
