# Supplementary material for: Household-level risk factors for Aedes aegypti pupal density in Guayaquil, Ecuador
Source: Parasit Vectors. 2021 Sep 7;14:458. doi: 10.1186/s13071-021-04913-0 (PMC8425057; doi:10.1186/s13071-021-04913-0)
Supplement: Supplementary file 2 — Additional file 2: Table S2. Full list of container-level candidate variables used to find the best model by AICc. [file 13071_2021_4913_MOESM2_ESM.docx]

Table S2. Full list of container-level candidate variables used to find the best model by AICc.

| Full List of Possible Model Variables | Variable Description |
| --- | --- |
| Outside location | The breeding site is within the walls of the house or outside the walls of the house |
| Contaminated water | Cloudy water or water containing particulates |
| Sewer part | Of or relating to a sewer, waterway, storm drain or manhole |
| Bucket part | Of or relating to a bucket or bucket cap |
| Bamboo | Natural material bamboo |
| Trash | Of or relating to trash (i.e. bag, Styrofoam box, cistern) |
| Toilet | Of or relating to toilets (i.e. indoor toilet, temporary toilet) |
| Small liquid container | Smaller artificial containers (i.e. flask, bottle, cup, jar, bowl, pot) |
| Tank | A tank used for storage or part of household utility system |
| Vase | A flower vase |
| Furniture | Household furniture items (i.e. table, drawer, chair, refrigerator) |
| Car part | A car fender or a wheel rim |
| Sink | Any appliance used as a sink (i.e. Outside sink, washbasin, laundry sink) |
| Pool | Large recreational area for swimming |
| Tub | Bathing tub |
| Barrel | Often used for water storage practices |
| Plastic material |  |
| Metal material |  |
| Cement Material |  |
| Rubber Material |  |
| Ceramic Material |  |
| Glass Material |  |
