# Supplementary material for: Household-level risk factors for Aedes aegypti pupal density in Guayaquil, Ecuador
Source: Parasit Vectors. 2021 Sep 7;14:458. doi: 10.1186/s13071-021-04913-0 (PMC8425057; doi:10.1186/s13071-021-04913-0)
Supplement: Supplementary file 3 — Additional file 3: Table S3. Variance inflation factors (VIF) that assess multicollinearity find that (outside of interaction terms) there are no VIF scores > 2. [file 13071_2021_4913_MOESM3_ESM.docx]

Table S3. Variance inflation factors (VIF) assess multicollinearity found that (outside of interaction terms) there are no VIF scores >2.

| Variable | VIF |
| --- | --- |
| Large solid collection | 1.519 |
| Unemployment | 1. 047 |
| Water volume | 1.250 |
| Canopy use | 1.519 |
| Precipitation at week 0 | 1.355 |
| Precipitation at week 2 lag | 14.225 |
| Large solid collection * Week 2 lag | 14.612 |
