# Supplementary material for: Household-level risk factors for Aedes aegypti pupal density in Guayaquil, Ecuador
Source: Parasit Vectors. 2021 Sep 7;14:458. doi: 10.1186/s13071-021-04913-0 (PMC8425057; doi:10.1186/s13071-021-04913-0)
Supplement: Supplementary file 4 — Additional file 4: Table S4. Full list of top candidate models for artificial breeding sites. Top models included have ∆AICc < 1. [file 13071_2021_4913_MOESM4_ESM.docx]

Table S4. Full list of top candidate models for artificial breeding sites. Top models included have ∆AICc < 1.

| Model | df | LogLik | ΔAICc |
| --- | --- | --- | --- |
| Car parts + Contaminated water + Furniture + Ceramic material + Glass material + Metal material + Plastic material + Sewer + Vase | 11 | -3459.19 | 0 |
| Car Parts + Contaminated water + Ceramic material + Glass material + Metal material + Plastic material + Sewer + Vase | 10 | -3460.23 | 0.03 |
| Bamboo + Car parts + Contaminated water + Furniture + Ceramic material + Glass material + Metal material + Plastic material + Sewer + Vase | 12 | -3458.20 | 0.08 |
| Bamboo + Car parts + Contaminated water + Ceramic material + Glass material + Metal material + Plastic material + Sewer + Vase | 11 | -3459.24 | 0.11 |
| Bucket part + Car parts + Contaminated water + Ceramic material + Glass material + Metal material + Plastic material + Sewer + Tub + Vase | 12 | -3458.28 | 0.24 |
| Bucket part + Car parts + Contaminated water + Ceramic material + Glass material + Metal material + Plastic material + Sewer + Vase | 11 | -3459.33 | 0.27 |
| Bamboo + Bucket part + Car parts + Contaminated water + Ceramic material + Glass material + Metal material + Plastic material + Tub + Vase | 13 | -3457.31 | 0.35 |
| Bamboo + Bucket part + Car parts + Contaminated water + Ceramic material + Glass material + Metal material + Plastic material + Vase | 12 | -3458.35 | 0/37 |
| Bucket part + Car parts + Contaminated water + Furniture + Ceramic material + Glass material + Metal material + Plastic material + Vase | 12 | -3458.39 | 0.45 |
| Bamboo + Bucket part + Car parts + Contaminated water + Furniture + Ceramic material + Glass material + Metal material + Plastic material + Vase | 13 | -3457.41 | 0.55 |
| Car parts + Contaminated water + Ceramic material + Glass material + Metal material + Plastic material + Sewer + Tub + Vase | 11 | -3459.49 | 0.60 |
| Bucket part + Car parts + Contaminated water + Furniture + Ceramic material + Glass material + Metal material + Plastic material + Sewer + Tub + Vase | 13 | -3457.43 | 0.60 |
| Bamboo + Car parts + Contaminated water + Ceramic material + Glass material + Metal material + Plastic material + Sewer + Tub + Vase | 12 | -3458.51 | 0.69 |
| Car parts + Contaminated water + Furniture + Ceramic material + Glass material + Metal material + Plastic material + Sewer + Tub + Vase | 12 | -3458.51 | 0.69 |
| Bamboo + Bucket parts + Car part + Contaminated water + Furniture + Ceramic material + Glass material + Metal material + Plastic material + Sewer + Tub + Vase | 14 | -3456.46 | 0.71 |
| Contaminated water + Cement material + Ceramic material + Glass material + Sewer + Vase | 8 | -3462.64 | 0.77 |
| Bamboo + Car parts + Contaminated water + Furniture + Ceramic material + Glass material + Metal material + Plastic material + Sewer + Tub + Vase | 13 | -3457.52 | 0.78 |
| Car parts + Contaminated water + Furniture + Cement material + Ceramic material + Glass material + Sewer + Vase | 10 | -3460.61 | 0.79 |
| Contaminated water + Furniture + Cement material + Ceramic material + Glass material + Sewer + Vase | 9 | -3461.67 | 0.86 |
| Car parts + Contaminated water + Cement material + Ceramic material + Glass material + Sewer + Vase | 9 | -3461.67 | 0.87 |
| Bamboo + Car parts + Contaminated water + Furniture + Cement material + Ceramic material + Glass material + Metal material + Plastic material + Sewer + Vase | 13 | -3457.58 | 0.88 |
| Bamboo + Car parts + Contaminated water + Cement material + Ceramic material + Glass material + Metal material + Plastic material + Sewer + Vase | 12 | -3458.62 | 0.91 |
| Bucket part + Contaminated water + Cement material + Ceramic material + Glass material + Sewer + Vase | 9 | -3461.73 | 1.00 |
