# Supplementary figures and images for: Household-level risk factors for Aedes aegypti pupal density in Guayaquil, Ecuador
Source: Parasit Vectors. 2021 Sep 7;14:458. doi: 10.1186/s13071-021-04913-0 (PMC8425057; doi:10.1186/s13071-021-04913-0)

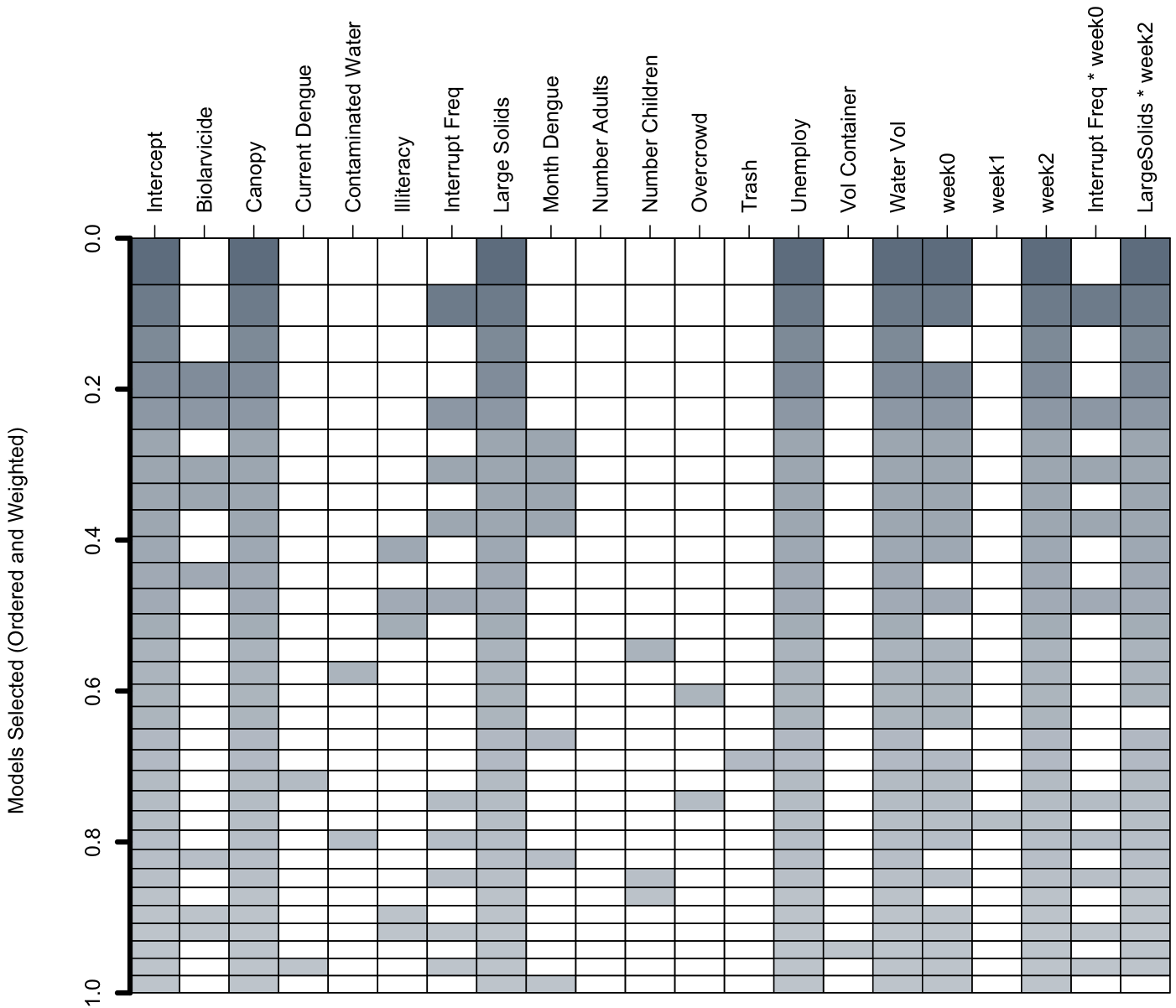

Supplement: Supplementary file 5 — Additional file 5: Figure S1. Model selection table where the top axis are all the candidate variables for pupal index (Additional file 1: Table S1) and the y-axis represents the frequency the variables were selected in the top candidate models with ∆AICc < 2 (31 models shown). [file 13071_2021_4913_MOESM5_ESM.tiff]

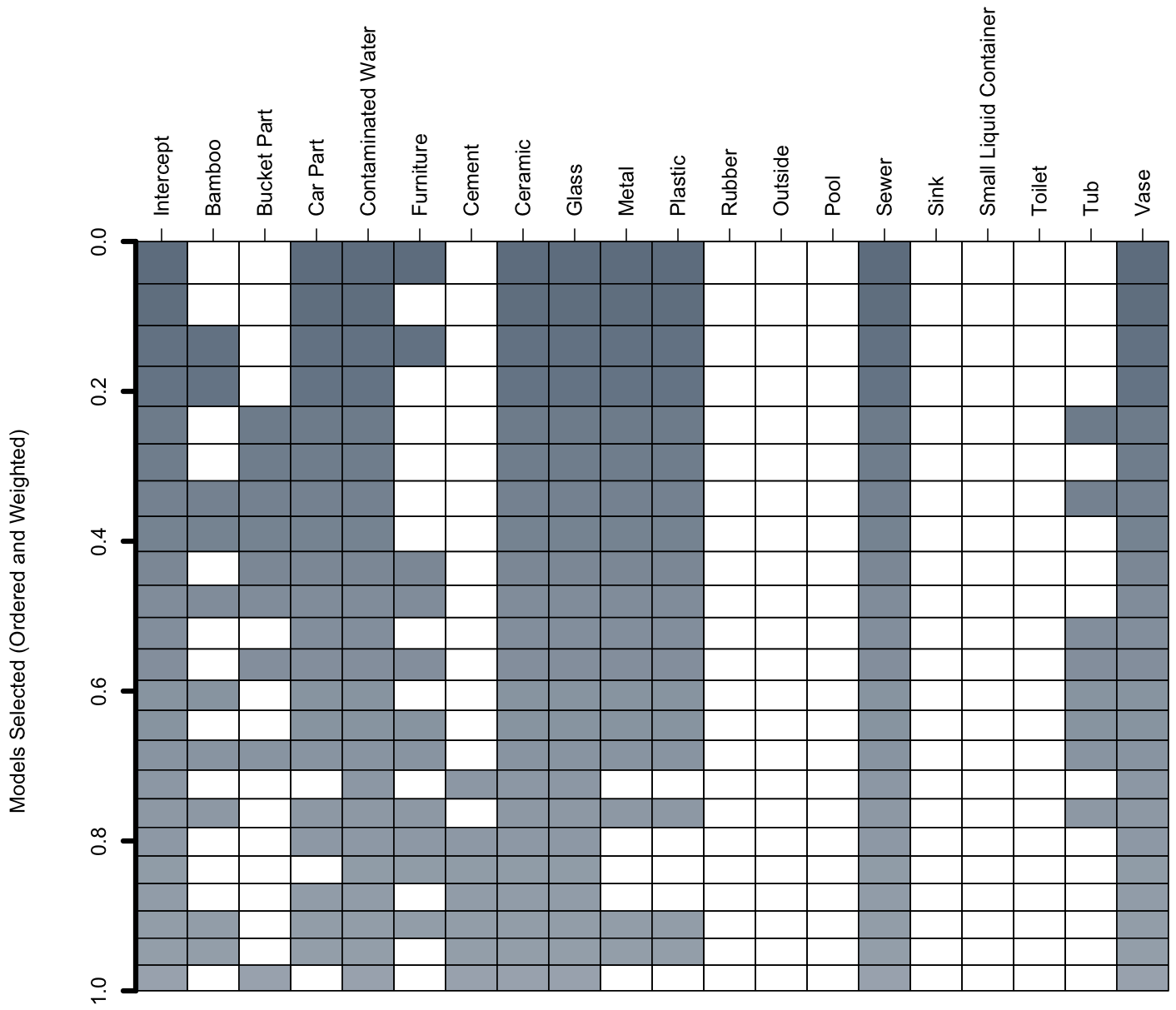

Supplement: Supplementary file 6 — Additional file 6: Figure S2. Model selection table where the top axis are all the candidate variables for pupal sum in containers (Additional file 2: Table S2) and the y-axis represents the frequency the variables were selected in the top candidate models with ∆AICc < 1 (23 models total shown). [file 13071_2021_4913_MOESM6_ESM.tiff]

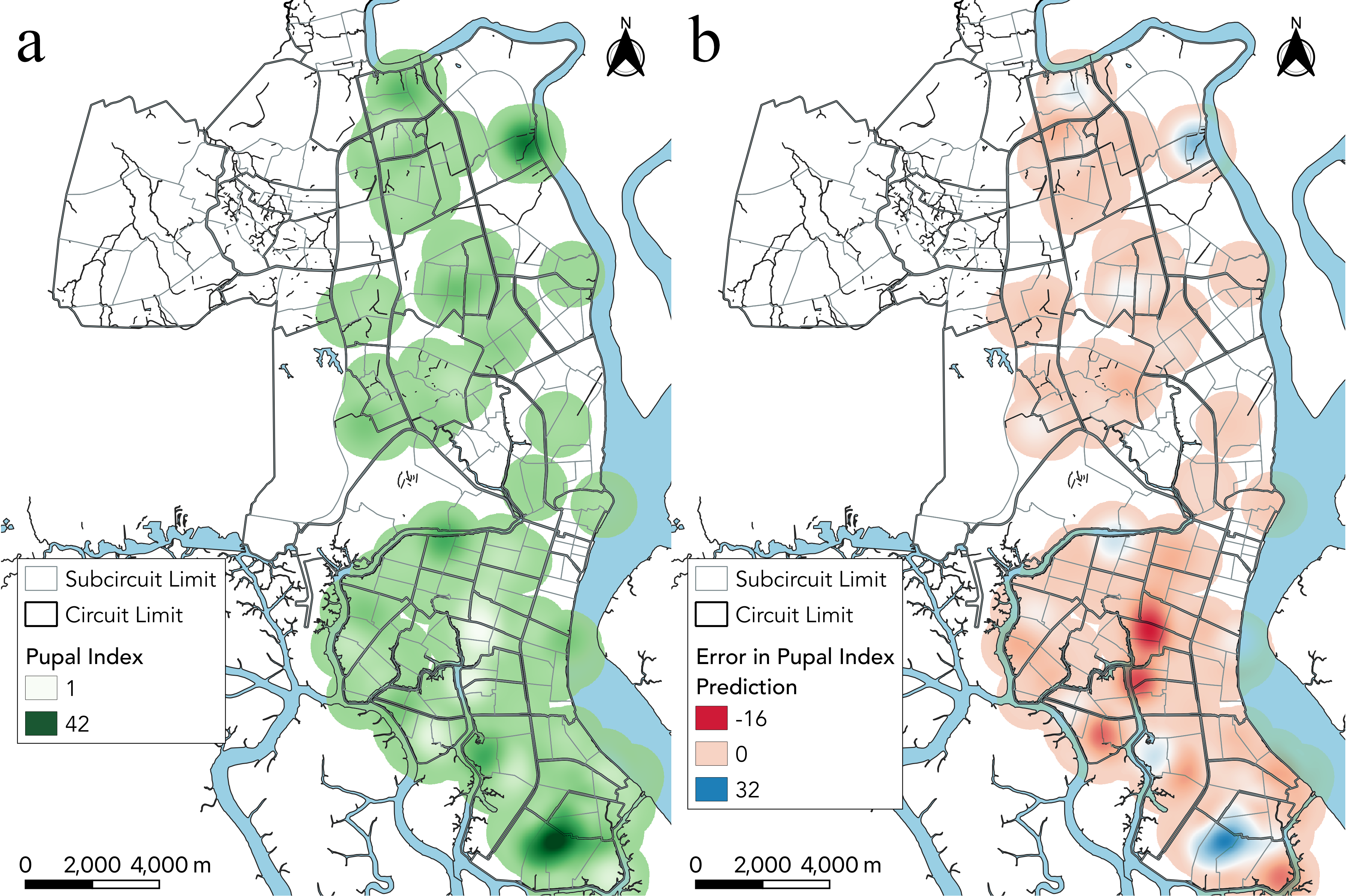

Supplement: Supplementary file 7 — Additional file 7: Figure S3. Pupal index measurements (a) and prediction error heat maps (b) based on the final model. Weighted by value with a bandwidth of 1 km. Error is the difference between the data and the model for each household’s characteristics. [file 13071_2021_4913_MOESM7_ESM.tiff]

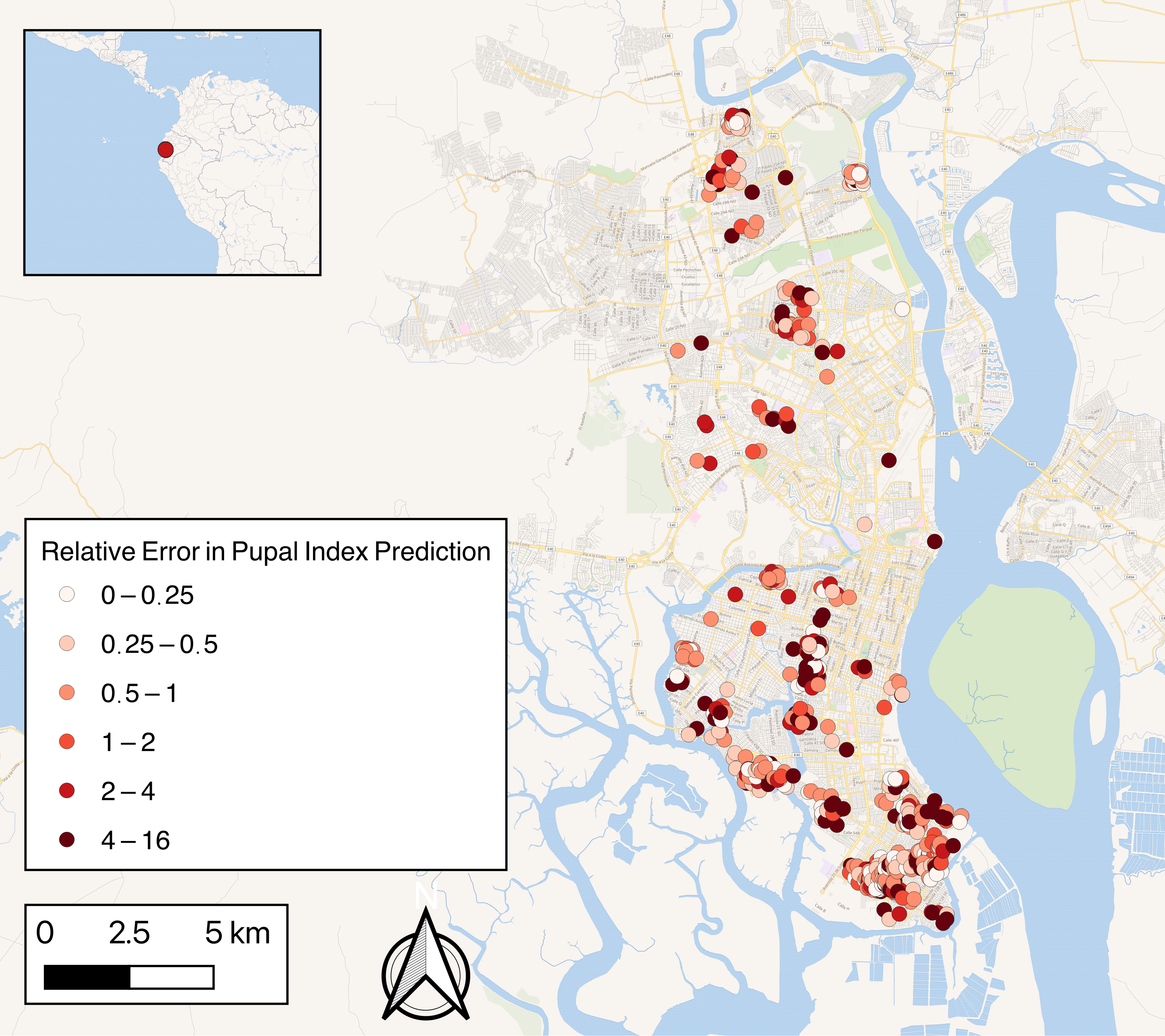

Supplement: Supplementary file 8 — Additional file 8: Figure S4. Relative error map for pupal index prediction based on the final model. Each dot indicates the relative prediction error of that household, i.e., the difference between data and model for each household’s characteristics divided by the data. [file 13071_2021_4913_MOESM8_ESM.tiff]
